# Editorial: Case reports in anesthesiology 2025

**DOI:** 10.3389/fmed.2026.1856090

**Published:** 2026-05-25

**Authors:** Ata Murat Kaynar

**Affiliations:** Department of Anesthesiology and Perioperative Medicine, University of Pittsburgh School of Medicine, Pittsburgh, PA, United States

**Keywords:** argatroban, arginine vasopressin deficiency, complex regional pain disorder, dexmedetomidine and delirium, respiratory distress

The case report remains one of medicine's most enduring vehicles for knowledge transfer. While randomized controlled trials set the standard of evidence, it is the single-patient or a small patient series narrative—unexpected, unscripted, and often humbling—that forces the clinician to revisit assumptions and refine practice. The case reports assembled here, spanning neuroendocrinology, regional anesthesia, hematology, coagulation medicine, neuromodulation, and emergency sedation, share no single organ system, yet they converge on three themes that are very much at the forefront of contemporary anesthesia and critical care: 1- the underestimated sequelae of seemingly routine procedures, 2- the imperative for individualized pharmacological decision-making, and 3- the expanding role of minimally invasive interventional techniques in populations once considered too complex or too frail for intervention.

Arginine vasopressin (AVP) after unruptured ACOM aneurysm clipping (Gundersen et al., *Frontiers in Endocrinology*, 2025) challenges the assumption that hypothalamic injury is the exclusive domain of ruptured aneurysms or major hemorrhagic events. Their 50-year-old patient developed frank AVP deficiency, adipsia, thermodysregulation, and clinically meaningful cognitive impairment following elective clip ligation of an 8 mm anterior communicating artery aneurysm—a procedure performed without intraoperative complication. MRI at 1 week revealed delayed infarction in the genu rostrum of the corpus callosum and bilateral edema of the anterior fornix columns, consistent with ischemia of perforating branches of the ACOM. A systematic literature review identified only seven prior cases of AVP deficiency in the setting of unruptured ACOM surgery, underscoring the rarity of the condition while simultaneously illustrating how easily it can be misattributed to post-operative delirium or perioperative fluid dysregulation. The case is a reminder that hypernatremia in the neurosurgical patient is not always a housekeeping problem. Adipsia compounded by cognitive impairment created a vicious cycle in which the patient lacked both the physiological drive to drink and the executive function to follow prescribed fluid regimens—a combination that poses an underappreciated risk for outpatient morbidity long after discharge. The authors advocate for prophylactic nimodipine consideration and, crucially, structured outpatient follow-up protocols that explicitly screen for osmolar dysregulation and thirst perception.

Dexmedetomidine nasal spray for hyperactive post-operative delirium (Liu et al., *Frontiers in Medicine*, 2025) addresses the opposite side of the post-operative neuropsychiatric spectrum. A 39-year-old man with no psychiatric history developed severe hyperactive delirium in the post-anesthesia care unit following emergency thoracoscopic surgery for rib fractures. Standard intravenous midazolam did control agitation briefly but precipitated oxygen desaturation due to respiratory suppression, a not uncommon risk in a patient breathing spontaneously after major thoracic surgery. A single 50 μg dose of intranasal dexmedetomidine resolved the delirium within 20 min and produced physiological NREM-like sleep without respiratory compromise, hemodynamic instability, or recurrence over the subsequent 7 days. The mechanistic rationale is well-grounded: dexmedetomidine's selectivity for presynaptic α2-adrenoceptors in the locus coeruleus suppresses norepinephrine release and disinhibits GABAergic neurons in the ventrolateral preoptic nucleus, mimicking natural sleep architecture in a way that benzodiazepines, with their broad GABA-A agonism and anticholinergic footprint, do not. The nasal route removes the practical barrier of intravenous access in an agitated patient who may be actively dislodging lines. This case joins a small but growing body of evidence supporting non-parenteral dexmedetomidine as both a preventive and—now—a therapeutic agent for post-operative delirium, and it points toward an important gap in current clinical guidelines, which remain largely silent on treatment once delirium is established.

High-molecular-weight kininogen deficiency and perioperative management (Jiang et al., *Frontiers in Medicine*, 2026) confronts a scenario increasingly likely to be encountered as genetic testing becomes routine: a young woman scheduled for laparoscopic-hysteroscopic surgery whose isolated markedly prolonged APTT (133.9 s) triggered a workup culminating in a homozygous KNG1 frameshift mutation—HMWK deficiency, with a global prevalence estimated near one per eight million. The paradox of HMWK deficiency is instructive: the contact activation system, though responsible for the prolonged *in-vitro* APTT that alarms everyone in the preoperative space, is bypassed entirely in physiological hemostasis, which proceeds through the tissue-factor pathway. Patients do not bleed abnormally; they may, however, be at modestly increased thrombotic risk due to impaired plasminogen activation. The authors navigated this with individualized risk stratification—thromboelastography to assess true clotting competence, fresh frozen plasma on standby given the novel mutation and endometrial surgical involvement, and early post-operative low-molecular-weight heparin rather than prophylactic hemostatic agents. The patient had minimal blood loss and was discharged on day four. The broader lesson is operational: a prolonged APTT that corrects immediately and at 2 h, with no factor deficiency on individual assay, should raise contact-pathway deficiency in the differential and trigger genetic testing rather than reflexive cancellation or empirical blood product administration.

Transient urinary retention after bilateral lumbar erector spinae plane block (Li et al., *Frontiers in Medicine*, 2025) documents the first reported case of sacral plexus blockade as a complication of lumbar ESPB. A 64-year-old man with failed back surgery syndrome at L5/S1 received bilateral ESPB at L5 for preoperative lumbosacral analgesia. Approximately 1 h later he was unable to void despite a strong urge; catheterization yielded 700 mL of urine, and spontaneous voiding returned the following morning as lumbosacral sensation recovered, timing that precisely mirrored the expected duration of ropivacaine block. The authors propose that prior fusion surgery created fascial scarring and disrupted anatomical planes, allowing the local anesthetic to track through the intervertebral foramen into the epidural space and reach the S2–S4 sacral parasympathetic outflow responsible for detrusor contraction. Notably, high injection resistance was observed intraoperatively—a warning sign that practitioners should recognize. ESPB has accrued an enviable safety record across thousands of reported cases, but the architecture of the lumbar fascial planes is more variable than its thoracic counterpart, and prior spinal surgery further unpredictably alters those planes. This case argues for routine injection-pressure monitoring, careful pre-procedural review of spinal imaging, and explicit post-operative monitoring of urinary function in patients with prior lumbosacral surgery.

Argatroban for intraoperative anticoagulation during left atrial appendage occlusion (Eappen et al., *Frontiers in Medicine*, 2025) tackles a narrow but clinically urgent problem: what to do when the standard anticoagulant is absolutely contraindicated and its usual alternative is itself problematic. The patient—a 67-year-old with two documented episodes of HIT type II and end-stage renal disease on hemodialysis—required LAAO with a Watchman device. Bivalirudin, the most familiar HIT-alternative in interventional cardiology, accumulates significantly in renal failure; its half-life extends from approximately 25 min in healthy adults to roughly 3.5 h on intermittent hemodialysis. Argatroban, hepatically cleared, has no dose adjustment for any degree of renal impairment. Using a 17% reduced initial bolus (290 mcg/kg rather than 350), the team found ACT rapidly exceeded 400 s—illustrating that uremic platelet dysfunction and baseline coagulopathy can substantially amplify argatroban's effect, and that iACT monitoring, while not formally validated for argatroban, was the practical point-of-care tool available. The procedure was completed without thrombotic or hemorrhagic complication. The case makes a practical case for pre-procedural multidisciplinary planning, conservative initial dosing, and serial ACT checks when argatroban is used outside its best-characterized indications.

Painless complex regional pain syndrome (Arefyeva et al., *Frontiers in Musculoskeletal Disorders*, 2025) describes a 32-year-old soldier who, following blast injury to the left lower extremity without penetrating trauma, developed progressive foot weakness, cyanotic discoloration, temperature asymmetry, and edema—the full autonomic and motor phenotype of CRPS—without pain. An exhaustive diagnostic battery, including nerve conduction studies, EMG, MEPs, SSEPs, MRI, isotope angiography, and Doppler ultrasound, excluded neuropathy, radiculopathy, and vascular occlusive disease, leaving CRPS as a diagnosis of exclusion. The case is paradoxical by definition: the Budapest Criteria, the gold standard since 2004, require continuing pain as a prerequisite for diagnosis. The authors note prior literature describing perhaps a hundred such painless CRPS-like presentations, including Eisenberg and Melamed's 2003 proposal of the term “complex regional painless syndrome.” Without pain to prompt the patient to seek care or to guide analgesic titration, the condition risks prolonged diagnostic delay and inappropriate management. Physical therapy and ankle-foot orthosis provided meaningful functional benefit where pharmacotherapy was not indicated.

CT-guided sequential neuromodulation for refractory chronic orchialgia rounds out the Research Topic with a case that exemplifies the translation of interventional pain techniques into geriatric medicine. A 68-year-old man with 5 years of neuropathic testicular pain, refractory to tamsulosin, NSAIDs, and escitalopram, achieved complete pain resolution (VAS 0) through a two-stage approach: diagnostic CT-guided hypogastric plexus block confirming the visceral pain pathway, followed by pulsed radiofrequency ablation of the superior hypogastric plexus. Complete pain freedom was sustained at 3 months. The case is notable for its adherence to a structured stepped-care model aligned with EAU 2025 guidelines, for its use of cross-sectional imaging guidance to navigate pelvic anatomy safely in an elderly patient with surgical contraindications, and for its implicit argument that age and comorbidity should not foreclose interventional options when pharmacological and conservative measures have been genuinely exhausted.

Taken together, these seven cases illuminate several convergent trends. First, procedural complications in anesthesia and surgery are increasingly being recognized as delayed, anatomically displaced, and physiologically mediated rather than immediately obvious at the time of intervention—the ACOM case, the ESPB case, and arguably the HMWK case all illustrate this. Second, pharmacological decision-making at the intersection of rare disease and organ dysfunction demands individualization that population-level dosing guidelines cannot fully anticipate; both the argatroban case and the HMWK case demonstrate how a well-reasoned, multidisciplinary, real-time pharmacological strategy can bridge that gap safely. Third, minimally invasive neuromodulatory and regional techniques are moving from rescue options of last resort to first-line considerations in carefully selected patients, particularly the elderly and those with multiple comorbidities, provided that anatomy is respected and complications are anticipated. Fourth, atypical presentations of well-characterized syndromes—painless CRPS, cognitively impaired AVP deficiency—continue to cause diagnostic delay precisely because the defining feature is absent, and broadening diagnostic criteria or creating explicitly recognized atypical categories may ultimately serve patients better than requiring full phenotypic expression. Clinicians willing to interrogate the unexpected, as each of these authors did, are the mechanism by which the medical literature translates rarity into readiness.

